# Polyamine Oxidase Is Involved in Spermidine Reduction of Transglutaminase Type 2-Catalyzed *β*H-Crystallins Polymerization in Calcium-Induced Experimental Cataract

**DOI:** 10.3390/ijms21155427

**Published:** 2020-07-30

**Authors:** Carlo Mischiati, Giordana Feriotto, Claudio Tabolacci, Fabio Domenici, Sonia Melino, Ilaria Borromeo, Cinzia Forni, Angelo De Martino, Simone Beninati

**Affiliations:** 1 Department of Biomedical Sciences and Surgical Specialties, University of Ferrara, 44121 Ferrara, Italy; msc@unife.it; 2 Department of Chemical and Pharmaceutical Sciences, University of Ferrara, 44121 Ferrara, Italy; frn@unife.it; 3 Department of Oncology and Molecular Medicine, Istituto Superiore di Sanità, 00161 Rome, Italy; claudiotabolacci@tiscali.it; 4 Department of Chemical Sciences and Technology, University of Rome “Tor Vergata”, 00133 Rome, Italy; fabio.domenici@uniroma2.it (F.D.); sonia.melino@uniroma2.it (S.M.); 5 Department of Physics, University of Rome “Tor Vergata”, 00133 Rome, Italy; ilaria18scv@hotmail.it; 6 Department of Biology, University of Rome “Tor Vergata”, 00133 Rome, Italy; forni@uniroma2.it (C.F.); Demartino@bio.uniroma2.it (A.D.M.)

**Keywords:** rabbit eye lens, cataract, TG2, spermidine, FAD-PAO, protein post-translation modification

## Abstract

In an in vitro Ca^2+^-induced cataract model, the progression of opacification is paralleled by a rapid decrease of the endogenous levels of spermidine (SPD) and an increase of transglutaminase type 2 (TG2, EC 2.3.2.13)-catalyzed lens crystallins cross-linking by protein-bound *N*^1^-*N*^8^-*bis*(γ-glutamyl) SPD. This pattern was reversed adding exogenous SPD to the incubation resulting in a delayed loss of transparency of the rabbit lens. The present report shows evidence on the main incorporation of SPD by the catalytic activity of TG2, toward *β*H-crystallins and in particular to the *β*B2- and mostly in *β*B3-crystallins. The increase of endogenous SPD in the cultured rabbit lens showed the activation of a flavin adenine dinucleotide (FAD)-dependent polyamine oxidases (PAO EC 1.5.3.11). As it is known that FAD-PAO degrades the *N*^8^-terminal reactive portion of *N*^1^-*mono*(γ-glutamyl) SPD, the protein-bound *N*^8^-*mono*(γ-glutamyl) SPD was found the mainly available derivative for the potential formation of *β*B3-crystallins cross-links by protein-bound *N*^1^-*N*^8^-*bis*(γ-glutamyl)SPD. In conclusion, FAD-PAO degradation of the *N*^8^-terminal reactive residue of the crystallins bound *N*^1^-*mono*(γ-glutamyl)SPD together with the increased concentration of exogenous SPD, leading to saturation of glutamine residues on the substrate proteins, drastically reduces *N*^1^-*N*^8^-*bis*(γ-glutamyl)SPD crosslinks formation, preventing crystallins polymerization and avoiding rabbit lens opacification. The ability of SPD and MDL 72527 to modulate the activities of TG2 and FAD-PAO involved in the mechanism of lens opacification suggests a potential strategy for the prevention of senile cataract.

## 1. Introduction

The majority of the dry weight of the eye lens is composed of soluble and long-lived proteins called crystallins [1]. In the mammalian lens, these proteins are divided into three major classes, *α*-, *β*-, and *γ*-crystallins, according to the size of their oligomers [2]. In particular, α-crystallins are a molecular chaperone as well as structural proteinsThey inhibit inflammation and subcellular transport of certain proteins, prevent cell death, provide neuroprotection, and regulate proteasomal degradation of proteins, whereas *β*- and γ-crystallins act only as structural proteins [3]. Native *β*-crystallins are oligomers, which elute in two or more size classes during gel-filtration, and range from 50–200 kDa. They contain seven different types of subunits named *β*B1, *β*B2, *β*B3 (basic) and *β*A1, *β*A2, *β*A3, *β*A4 (acidic) which associate to form polymers with low (*β*L) or high molecular weight (*β*H) [4]. Lens crystallins have the remarkable ability to exist in high concentrations in the transparent lens, without causing light scatter. Crystallins possess a high degree of packing regularity, leading to optical properties responsible for lens transparency. Changes in the crystalline structure, degradation, and other chemical modifications lead to the insolubilization and aggregation of these proteins resulting in cataract [5], which represents the major cause of blindness worldwide. In particular, it is well established that in some cases the lens opacification process may depend on post-translational modification in various proteins, including crystallins [6].

TG2, which belongs to the transglutaminase family, is a multifunctional enzyme with transamidant, isomerase protein disulfide, and GTP/ATPase activity [7].

The transamidant activity of TG2 is the most well-characterized enzymatic activity. Indeed, among different types of post-translational modifications, the acyl transfer reaction between the γ-carboxamide group of glutamine residues and the ε-amino group of lysine residues mediates by transglutaminase 2 (TG2; EC 2.3.2.13) is noteworthy [8,9,10,11]. Naturally occurring di- and polyamines, putrescine (PUT), spermidine (SPD), and spermine (SPM) are excellent substrates for TG2 in vitro [12]. These amines may be incorporated into a glutamyl residue of a substrate protein with consequent formation of either *N*-*mono*(γ-glutamyl)- or *N*,*N*-*bis*(γ-glutamyl)-PUT, -SPD, or -SPM. In particular, SPD is an asymmetric molecule, with two primary and one secondary amino groups. It possesses a structure composed of an amino- butyl and an amino-propyl portion. The link with the glutamine residue is carried out on the amino group in position 1 or the amino group in position 8. The final products of this reaction, catalyzed by TG2 are *N*^1^-*mono*(γ-glutamyl)SPD and the *N*^8^-*mono*(γ-glutamyl)SPD. The free amino groups of both the derivatives can react with another glutamine residue, forming a cross-link with a bridge of *N*^1^,*N*^8^-*bis*(γ-glutamyl)SPD between the substrate proteins. Therefore, polyamines could exert a controlling influence on protein cross-linking, by competing against the ε-(γ-glutamyl) lysine residues [13]. High levels of polyamines favor the formation of *mono*-derivatives, due to the rapid saturation of the available reactive glutaminyl protein residues which cannot be involved in the formation of protein cross-links, or rather ε-(γ-glutamyl)lysine or *bis*(γ-glutamyl) derivatives. We have previously reported the modulatory effect of SPD, on TG2 activity, during in vitro rabbit eye lens opacification. It was possible to observe, in a calcium-induced cataract model, a rapid decrease of the endogenous levels of lens SPD, along with the progression of opacification, paralleled by an increase of crystallins cross-linking by *N*^1^,*N*^8^-*bis*(γ-glutamyl)-SPD [14]. The loss of transparency of lens was reversed, increasing the levels of lens SPD. In this condition, it was evidenced by the presence of an increased amount of both the *mono*(γ-glutamyl)SPD derivatives. Interestingly, a degradative pathway of γ-glutamyl-polyamines mediated by flavin adenine dinucleotide FAD-PAO has been previously described [15]. In the present investigation, pieces of evidence were collected showing that the post-translational modification of *β*H-crystallins, catalyzed by TG2 is modulated by the levels of lens SPD and regulated by the activity of FAD-PAO, through the degradation of one of the two TG2-formed *mono*-SPD derivatives, essential for polymerization of *β*H-crystallins.

## 2. Materials and Methods

### 2.1. Materials

Spermidine-tetrahydrochloride (SPD), guinea pig liver TG2 (GPL-TG2), standard crystallins from bovine eye lens, trichloroacetic acid (TCA), dithiothreitol (DTT), *ortho*-phthalaldehyde (OPA), dansylchloride, trifluoroacetic acid (TFA), acetonitrile (C_2_H_3_CN), MDL 72527 (N,N′-Bis(2,3-butadienyl)-1,4-butanediamine dihydrochloride) and all reagents were from Sigma-Aldrich (St. Louis, MO, USA). [^3^H]-SPD (18 Ci/mmol) were obtained from NEN Radiochemicals (Italy). *N*^1^-*mono*(γ-glutamyl)SPD, *N*^8^-*mono*(γ-glutamyl)SPD, *N*^1^-*N*^8^*bis*-(γ-glutamyl)SPD were generously provided by J. E. Folk (National Institutes of Health, Bethesda, MD, USA). Pronase, collagenase, aminopeptidase M, carboxypeptidases A, B, and Y were obtained from Boehringer, (Mannheim, Germany). Usual laboratory chemicals were purchased from Merck (Darmstadt, Germany).

### 2.2. Lens Culturing

The fresh rabbit eyes, obtained from a local slaughterhouse, were washed in concentrated ethanol for a few seconds. The cornea was dissected aseptically, the lens was removed free from residues of the ciliary body, zonular and vitreous fibers, washed in phosphate buffer saline (PBS) and cultivated and treated as previously described [14]. The transparency of all cultured lens groups has been checked and normalized to 100% transmittance. Treatments of the rabbit lens to inhibit FAD-PAO activity were performed by incubating the lens for 72, 96, and 120 h in the culture medium containing 400 μM MDL 72527.

### 2.3. Separation of Rabbit Lens Crystallins by Gel Permeation and SDS-PAGE

Briefly, lenses were decapsulated and homogenized in 10 mM Tris-HCl pH 7.5 containing 10 mM DTT and mixture of protease inhibitors: 1 µg/mL leupeptin, 1 µg/mL pepstatin, 1 mM bestatin and 0,1 mM PMSF. Lens homogenates were ultracentrifuged (50,000× *g*, 25 min, 4 °C) and supernatants, containing soluble crystallins, were isolated. For the gel permeation chromatography, the supernatant was adjusted to give a concentration of proteins of about 30–50 mg/mL. Gel permeation chromatography was carried out on Sepharose 6B (1.5 × 60 cm) column equilibrated with a buffer containing Tris-HCl 50 mM pH 9, NaCl 50 mM, and EDTA 1 mM. Relative molecular masses of the eluted fractions were determined using purified standard proteins (molecular weights 800–1000 kDa, 100–200 kDa, 50–100 kDa, and 20 kDa, respectively). Crystallins were also subjected to sodium dodecyl sulfate-polyacrylamide gel electrophoresis (SDS-PAGE) using a 10% polyacrylamide gel.

### 2.4. Incorporation of [^3^H]-SPD into Lens Crystallins from Gel-Permeation by Purified GPL-TG2

Gel-permeation fractions of *β*H-crystallins, after dialysis in ammonium bicarbonate and lyophilization, were dissolved in a buffer containing 10 mM DTT and 8M urea. Covalent incorporation of [^3^H]-SPD was carried out in an incubation medium containing 50 mM Tris-HCl (pH 7.4), 0.15 M NaCl, 1 mM DTT, 2.5 mM CaCl_2_, 5µL (5 µCi) of [^3^H]-SPD and GPL-TG2 (4 µg/mL). Controls were run at the same time in the presence of EDTA. After 2 hrs of incubation, the reactions were stopped with 1 mL of 10% TCA containing 2 mM unlabeled SPD. The samples were then centrifuged at 12.000× *g* for 10 min. The precipitates were washed twice with the solution described above and finally dissolved in 1 mL of 0.1 N NaOH. Aliquots (100 µL) from the dissolved precipitates were counted in 10 mL of ACS scintillation cocktail (Amersham). The saturation curve for the self-incorporation of SPD into GPL-TG2 was considered as a control. Radioactivity of samples was evaluated by a scintillation counter (Beckman LS-5000TD, Brea, CA, USA).

### 2.5. RP-HPLC Chromatography of βH-Crystallins

Protein fraction obtained by gel filtration chromatography (0.5 mg) corresponding to the *β*H-crystallins after dialysis in ammonium bicarbonate and lyophilization, was dissolved in 100 μL of 8 M urea and 10 mM DTT and in injected into RP-HPLC system (System Gold, Beckmann) using a C8 column (250 × 4.6 mm 7 mm Aquapore OD-300 Brownlee) and the following linear gradient of solvent B (solvent A 0.2% TFA v/v; solvent B 70% C_2_H_3_CN and 0.2% TFA *v*/*v*): 2 min at 0% solv. B, 0–40% solv. B in 40 min, after 5 min 40–70% solv. B in 50 min and after 5 min 70–90% solv. B in 10 min, the flow rate was 2.5 mL/min. The absorbance was monitored at λ = 220 nm. Covalent incorporation of [^3^H]-SPD into the seven HPLC fractions of rabbit lens crystallins, was carried out as described in the previous paragraph.

### 2.6. N-terminal Amino Acids Sequencing of βH-Crystallins

Samples (0.3–0.6 nmol) were loaded onto methanol-preconditioned PVDF membranes. The N-terminal sequence of the three chromatographic peaks was not available by simple NH_2_-terminal sequence analysis using Edman degradation, because they were NH_2_-terminally blocked. Therefore, the *β*H subunits were spotted on the PVDF membrane and cleaved at Met residues by reaction with CNBr in 70% formic acid overnight at room temperature. The N-terminal amino acid sequence of the fragments after the reaction was then performed by automated Edman degradation using an Applied Biosystems gas phase sequencer model 473A with a PTH amino acid derivative identification.

### 2.7. Determination of Endogenous Free SPD and Its (γ-Glutamyl) Derivatives

Levels of free SPD in cultured rabbit lens were evaluated as described [16]. Isolation and quantification of γ-glutamyl-derivatives of SPD were performed by preliminary proteolytic digestion of lens protein pellets [13]. Chromatographic separation of *mono*- and -*bis*(γ-glutamyl)SPD was carried out by an HPLC procedure as previously reported [17].

### 2.8. FAD-PAO Activity Assay

FAD-PAO activity in rabbit lens homogenates was measured by a modified spectrophotometric method using SPM-tetrahydrochloride as substrate [18,19].

### 2.9. Transglutaminase Activity Assay

Analysis of the transamidant activity of TG2 in rabbit lens homogenates was performed by a Fluorescent Transglutaminase Assay (FTA-Zedira GmbH). TG2 catalyzes acyl transfer reactions from glutamine residues in proteins or peptides to primary amines. The TG2-catalyzed covalent coupling of *mono*-dansylcadaverine into *N*,*N*-dimethylcaseine produces a shift in intensity and wavelength of fluorescence of the dansyl group. The TG2 activity can be monitored online by measurement of the fluorescence (excitation wavelength *λ* = 332 nm; emission wavelength *λ* = 500 nm).

### 2.10. Lens Transparency Evaluation

All cultured lens groups (control, SPD, and MDL 72527-treated) were homogenized in lysis buffer (50 mM Tris-HCl, pH 8.0; 150 mM NaCl, 1% Igepal and a mixture of protease inhibitors). The absorbance at *λ* = 350–420 nm for each sample was measured at the spectrophotometer. The transparency was calculated as a function of transmittance, according to Jain and colleagues [20]. The data are expressed as relative absorbance at *λ* = 394 nm.

Lens transparency was calculated as a function of transmittance, as previously reported [14].

### 2.11. Statistical Analysis

All experiments were repeated three times, and the results are expressed as the mean ± SD of three different determinations. Data were analyzed by the *t*-Student test, differences were considered significant when *p* < 0.05.

## 3. Results

### 3.1. Separation of Crystallins by Gel-Filtration and Identification by SDS-PAGE Analysis

**Figure 1:**
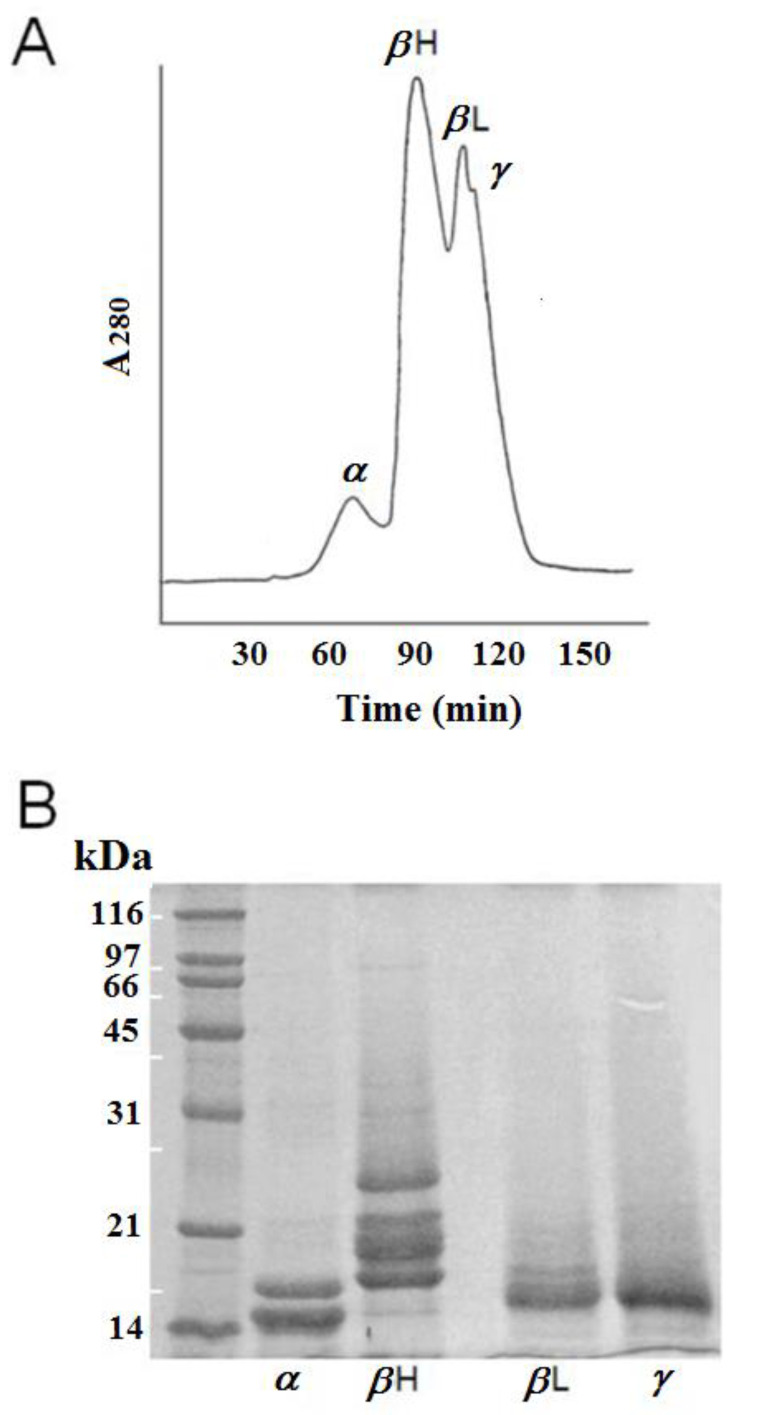
(**A**) Isolation and fractionation of rabbit lens crystallins by gel-permeation chromatography. Column eluates were monitored for absorbance at *λ* = 280 nm. The four crystallin fractions, labeled above each peak correspond to α, *β*H, *β*L, and γ-crystallins, respectively. The percentage yields for each crystallin class were estimated from the areas under each crystallin peak. (**B**) Gel electrophoresis of gel-permeation fractionated rabbit lens crystallins under denaturing conditions (SDS-PAGE) in the presence of 5 mM dithiothreitol. The gel was stained with Coomassie blue. The reference molecular weight standards were: *β*-galactosidase (116.3 kD), 97k-GP glycoprotein (97 kD), bovine serum albumin (66 kD), ovalbumin (45 kD), carbonic anhydrase (31 kD), soybean trypsin inhibitor (21.5), and lysozyme (14.4 kD).

Figure 1A shows the chromatographic profile, by gel-filtration, of rabbit lens crystallins divided into four classes as described previously [2]. This separation into α-, *β*H-, *β*L-, and γ-crystallins were confirmed by SDS-PAGE (Figure 1B). The native molecular masses of 4 eluted peaks were estimated from the column calibration with standard proteins (data not shown). The characteristic and well-separated patterns of doublet bands for α-crystallin (Figure 1B) are clearly shown by the SDS-PAGE analysis, similar to other mammalian α-crystallin fractions [21]. Moreover, the polypeptide composition of crystallins as determined by SDS-gel electrophoresis, revealed five high molecular weight bands for *β*H, two for beta *β*L, and one for γ crystallins (Figure 1B).

### 3.2. Transglutaminase-Catalyzed Incorporation of Polyamines into Crystallins

**Figure 2:**
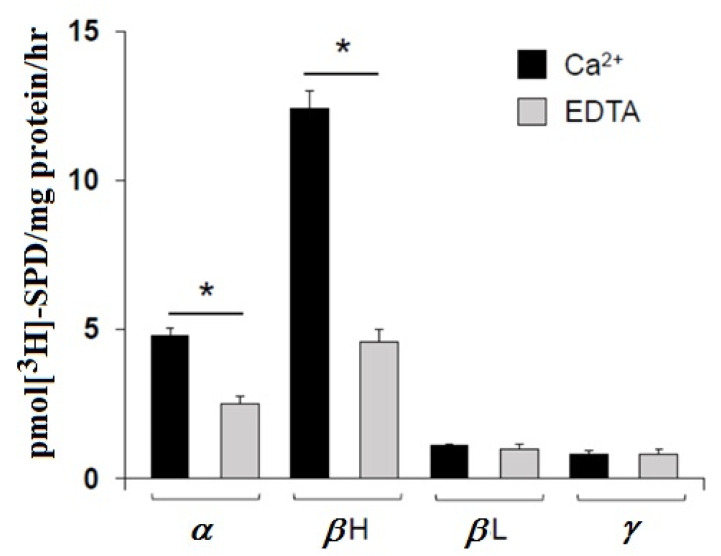
GPL-TG2-catalyzed incorporation of [^3^H]-SPD by rabbit eye lens crystallins separated by gel-permeation chromatography. Pellets derived from trichloroacetic acid (TCA)-precipitated protein were extensively washed, solubilized, and counted for radioactivity. EGTA incubation was used as a negative control. The data are expressed as the mean of three different determinations ± SD (* *p* < 0.05).

Gel-permeation fractions of rabbit lens crystallins, after dialysis in ammonium bicarbonate and lyophilization, were analyzed for the potential in vitro covalent incorporation of [^3^H]-SPD catalyzed by a purified TG2 from GPL-TG2. By this procedure, it was possible to assess the uptake of this polyamine into the lens proteins, and, above all, its TG-catalyzed incorporation into lens crystallins. The result, of these experiments, is summarized in Figure 2. The data suggest that *β*H-crystallins are the best substrate for TG2. The incorporation of radiolabeled SPD was less evident for α-crystallins and negligible for *β*L- and γ-crystallins. The specificity of the incorporation was further verified, measuring the amount of radioactivity in the lens protein by the inhibition of the activity of TG2 enzyme with EGTA (Figure 2).

### 3.3. HPLC Separation of βH-Crystallins and Identification of TG2 Substrates

**Figure 3:**
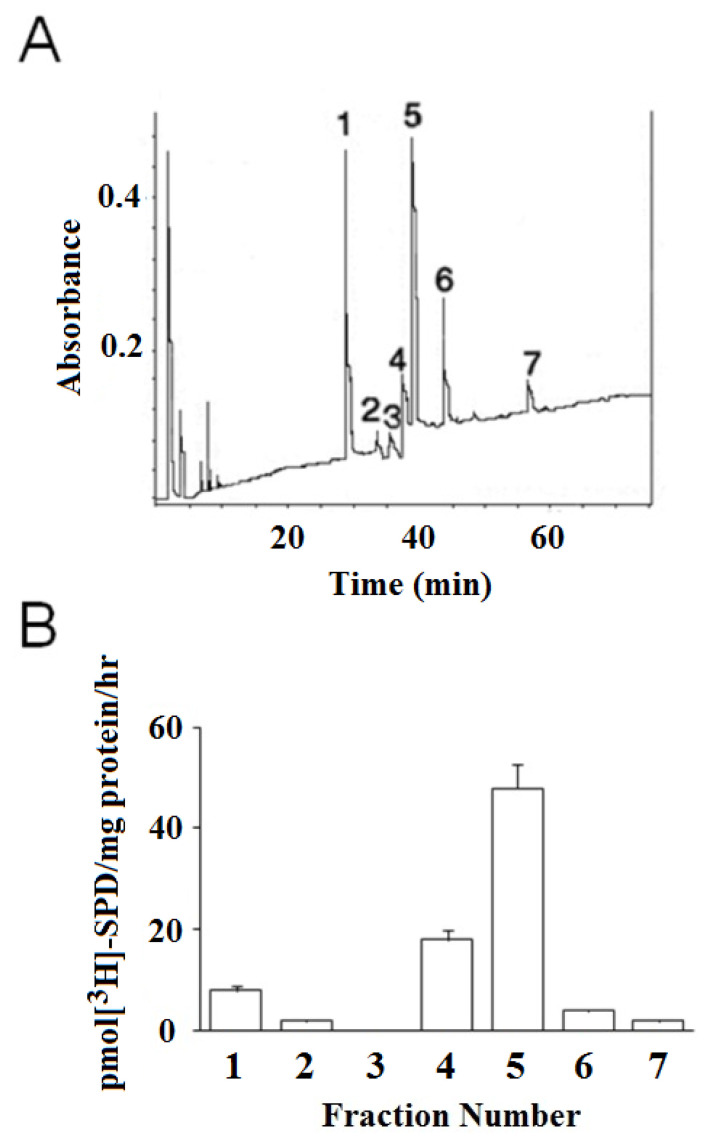
(**A**) HPLC chromatogram of the gel-permeation fraction corresponding to *β*H-crystallins. The detailed HPLC conditions are described in the Materials and Methods section. (**B**) GPL-TG2-catalyzed incorporation of [^3^H]-SPD by the chromatographic subunits from RP-HPLC separation of the *β*H-crystallins. The data are expressed as the mean of three different determinations ± SD.

HPLC separation resolved *β*H-crystallins into seven main sub-fractions (Figure 3A). Identification of TG2 substrates, in these chromatographic fractions of *β*H-crystallins, was carried out incubating each single HPLC fraction with [^3^H]-SPD and GPL-TG2 (Figure 3B). Among the resolved fractions, only the peaks marked as peak 1, 4, and 5 contained bound radioactivity. Particularly high was observed for peak 5. The sequence of these three chromatographic peaks, could not be identified by simple NH_2_-terminal sequence analysis, because they were NH_2_-terminally blocked. Therefore, the identification was performed by N-terminal sequencing analysis of the peaks after fragmentation at methionine residues by incubation with CNBr in acid conditions. The sequences obtained are shown in Supplementary Table S1 and the analysis of the sequence similarity confirmed that their identity was *β*B2- (peak 1), *β*B3- (peak 5), and a fragment of *β*B3-crystallin (peak 4), (Supplementary Figure S1).

### 3.4. Levels of the TG2 Catalyzed, SPD Derivatives, Linked to the Crystallins of Cultured Rabbit Lens, in Presence of Exogenous SPD

Gel-permeation fractions of *β*H-crystallins of rabbit lens cultured in the presence of 20 mM Ca^2+^ with and w/out 100 mM SPD, were subjected to proteolytic digestion as described in Materials and Methods. The γ-glutamyl-derivatives of SPD from endogenous TG2-catalyzed transamidation of rabbit lens proteins were determined by RP-HPLC chromatographic separation [17]. Table 1 shows the γ-glutamyl-SPD derivatives found in lens cultured for 72, 96, and 120 h, in the absence or in the presence of exogenous SPD during the time of incubation. A time-dependent decreased levels of protein-bound *N*^1^- and *N*^8^-*mono*(γ-glutamyl)SPD derivatives (from 50.3 ± 3.5 to 22.6 ± 9.2 pmol/mg protein for *N*^1^ and from 12.3 ± 0.5 to 6.7 ± 0.2 pmol/mg protein for *N*^8^) was observed in the absence of exogenous SPD. The decrease of these two *mono*-derivatives appears to be responsible for the observed increased cross-links of lens-crystallins by *N*^1^*,N*^8^*-bis*(γ-glutamyl)SPD (from 65.7 ± 8.4 to 125.4 ± 14.9 pmol/mg protein) followed by a remarkable decrease of lens transparency from 40% to 10% (Table 1). On the contrary, lens cultured in 100 mM SPD, showed evident time-dependent increased levels of protein-bound *N*^8^-*mono*(γ-glutamyl) SPD derivative (from 37.8 ± 2.5 to 87.7 ± 5.7 pmol/mg protein). In this condition *N*^1^-*mono*(γ-glutamyl)SPD was found decreased during the time of incubation (from 28.7 ± 1.5 to 15.3 ± 0.7 pmol/mg protein). The transparency of the cultured lens under these conditions has been assessed at 80% compared to 10% of the control, likely due to the remarkable reduction of *N*^1^*,N*^8^*-bis*(γ-glutamyl)SPD derivative formation (from 50.5 ± 2.5 to 17.4 ± 0.4 pmol/mg protein).

### 3.5. FAD-PAO and TG2 Activity in Rabbit Lens

**Figure 4:**
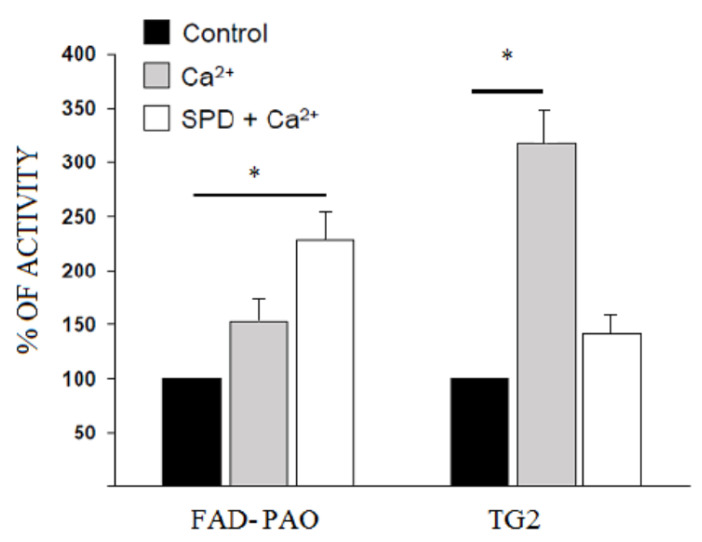
The activity of two enzymes involved in maintaining the transparency of the rabbit lens. flavin adenine dinucleotide-dependent polyamine oxidases (FAD-PAO) and TG2 were analyzed in the rabbit lens grown according to the experimental protocol (see Materials and Methods). The data are expressed as the mean of three different determinations ± SD (* *p* < 0.05).

The activity of the two enzymes involved in the opacification of the rabbit lens (FAD-PAO and TG2) was evaluated under the two experimental conditions of lens culturing, i.e., in the presence of Ca^2+^ alone or the presence of Ca^2+^ and SPD. As shown in Figure 4, Calcium activated TG2, more than three times respect to the control, while the presence of SPD reduced the activity of this enzyme, probably due to competitive inhibition of this polyamine with the standard substrate. Furthermore, the increased levels of SPD in the lens culture medium enhanced the activity of the FAD-PAO by more than two times (Figure 4).

### 3.6. Levels of the TG2-Catalyzed SPD Derivatives, in the Proteolytic Digest of Cultured Rabbit Lens upon PAO Activity Inhibition

The observed drastic reduction of the *N*^1^-*mono*(γ-glutamyl)SPD levels observed in the lens incubated in the presence of SPD as opposed to *N*^8^-*mono*(γ-glutamyl)-SPD levels (Table 1), led to the assumption of a possible degradation mechanism of the *N*^1^-*mono* derivative. Based on a previous experience [15] that showed that the *N*^1^-*mono* derivative of SPD is an excellent substrate of the FAD-PAO, the possible role of this enzyme in this phenomenon was investigated. Since rabbit lens FAD-PAO activity, in the presence of SPD was found increased (Figure 4) lenses were cultured with a FAD-PAO inhibitor, the MDL 72527 [22,23], exogenous SPD and Ca^2+^ as reported. Table 2 shows that the inhibition of PAO by MDL 72527, added to the medium of the cultured rabbit lens at a different time of incubation, leads to a time-dependent accumulation of *N*^1^-*mono*(γ-glutamyl) SPD (from 25.5 ± 4.5 to 65.3 ± 6.8 pmol/mg protein at 72 h of incubation and from 14.2 ± 0.4 to 88.7 ± 3.2 pmol/mg protein at 120 h of incubation. The *N*^8^-*mono*(γ-glutamyl) SPD levels were found unaffected by the MDL 72527 treatment. In conclusion, the presence of this FAD-PAO inhibitor, results in an accumulation of *N*^1^-*mono*(γ-glutamyl)SPD, with an evident decrease of *N*^1^,*N*^8^*-bis*(γ-glutamyl)SPD, responsible for the cross-linking of crystalline. In the presence of high levels of the two *mono*-derivatives, it is conceivable that the saturation of the glutamine protein residues necessary for the formation of the *bis*-derivate is being reached.

## 4. Discussion

Eye lens, which is highly protein-rich, is a tissue where protein turnover occurs over several decades in time. Modification of the protein’s structure is thought to lead to lens opacification or cataract [24]. An increase in the intracellular level of free Ca^2+^ is seen in the lens during aging and in cataract [25]. These increased Ca^2+^ levels appear to trigger the activity of some otherwise latent enzymes. Important among them are Ca^2+^-dependent calpain and TG2. TG2 has attracted attention after the isolation of ε-(γ-glutamyl)lysine isopeptide cross-linked products from the cataract-bearing lens [26]. TG2 catalyzes acyl-transfer reactions by which such isopeptide cross-links are formed, between glutamine and lysine residues of proteins and polypeptides [27]. TG2 acts solely on glutamine residues, which function as an acceptor substrate but also display high specificity for donor lysine substrate and biogenic polyamines [28]. The dimerization pathway of two proteins by polyamines, in a reaction catalyzed by TG2, can be divided into two steps. Initially, a primary amine group of a polyamine reacts with a γ-glutamyl site of the protein, leading to the formation of a *mono*(γ-glutamyl)-conjugate. The end product of this reaction relies on the chemical structure of the polyamine involved in the reaction. As PUT and SPM are symmetric molecules, their side of attachment to the protein is not peculiar. Therefore, the end product of the mono-conjugation of PUT or SPM to proteins is always an *N*-*mono*(γ-glutamyl)derivative. SPD derives from the condensation of a propyl moiety to putrescine, in a reaction catalyzed by SPD-synthase [29]. Since SPD is an asymmetric molecule, the TG2 conjugation to a protein leads to two possible products, i.e., *N*^1^-*mono*(γ-glutamyl)SPD and *N*^8^-*mono*(γ-glutamyl)SPD. Finally, the remaining primary amine group is cross-linked to another γ-glutamyl site [30].

Little is known about the natural steric arrangement of the protein post-translationally modified by polyamines, in any of the biologically occurring polymeric ensembles. No methods are available for disassembling γ-glutamyl-polyamine cross-linked structures, without destroying the backbones of the constituent units themselves. Several pathological conditions influenced by this irreversible post-translational protein modification have been reported [9]. The increased intra-lens concentration of Ca^2+^ has been used to induce cataract formation in animal studies and in vitro models, using lens organ culture or cell culture, though the mechanism for Ca^2+^-induced cataract is not fully understood [31]. In this study, explanted rabbit lenses were cultured in the presence of 20 mM CaCl_2_, the optimum concentration to induce opacification, and the soluble eye lens proteins were operationally defined into four fractions termed α, *β*H, *β*L, and γ crystallins. Recently, several different methods for the fractionation of the soluble lens proteins have been developed. Some of these methods have concerned about the preparation of all four classes of crystallins [24]. However, precise information concerning the number of structurally distinct proteins in each crystallin, their stoichiometry, and the nature of their interactions has not been obtained yet. Because of the complex nature of the crystallins, some investigators have deemed it desirable to secure initial fractionation of the soluble lens proteins into the four classical fractions (α, *β*H, *β*L, and γ crystallins), before undertaking detailed physicochemical studies of these proteins. To be meaningful, such studies require preparative procedures providing clear separation and quantitative recovery of each crystallin. Several of the recently reported methods for the preparation of soluble lens proteins have been evaluated by us for this purpose. The ability of these methods to fulfill the required criteria successfully is quite variable. Therefore, we have developed a scheme for achieving the desired fractionation utilizing gel-filtration and RP-HPLC.

The in vitro incorporation of [^3^H]SPD, catalyzed by GPL-TG2 of the four gel-permeation fractions, confirmed *β*H-crystallins as the best substrate for the enzyme. α–Crystallins were less reactive as substrates of GPL-TG2, and neither *β*L nor γ-crystallins formed appreciable amounts of cross-linked structures. The isolated gel-permeation *β*H-fraction was then analyzed through RP-HPLC and evidenced the presence of seven chromatographic peaks, of which only three of them were able of incorporating [^3^H]SPD in the presence of GPL-TG2. The identification, performed by sequencing tryptic fragments of the three chromatographic fractions, showed the presence of *β*B2, *β*B3, and a fragment of degraded *β*B3. Among these, *β*B3 incorporated most of [^3^H]SPD, proving to be an excellent substrate for GPL-TG2. To investigate the type of post-translational modification of lens *β*B3-crystallines induced by endogenous lens-TG2 in the presence of Ca^2+^ and cold SPD, a subsequent investigation was carried out, focusing on the formation of γ-glutamyl-derivatives of SPD. The rabbit lens, grown over a long period of time, in the absence of exogenous SPD, showed the formation of a high percentage of protein cross-links, evidenced by the formation of *N*^1^-*N*^8^-*bis*(γ-glutamyl)SPD, followed by a more pronounced lens opacification. In contrast, the increased concentration of exogenous SPD in the culture medium, confirmed a drastic reduction in protein cross-links, with significantly reduced lens opacification [14]. In this experimental context, the presence of exogenous SPD led to the drastic reduction of *N*^1^-*mono*(γ-glutamyl)SPD compared to *N*^8^-*mono*(γ-glutamyl)SPD derivative. These conflicting results, together with the observed low levels of *N*^1^-*N*^8^-*bis*(γ-glutamyl)SPD crosslinks were difficult to explain. Probably, the high concentration of exogenous SPD causing an increase of the *N*^8^-*mono*(γ-glutamyl) SPD derivative could saturate the γ-glutamyl residues of the substrate proteins, thus avoiding the formation of cross-links. This hypothesis appears highlighted by lens grown in the absence of SPD, where the formation of the cross-links reaches high concentrations with consequent opacification of the organ. It was also necessary to find evidence justifying the drastic reduction in the *N*^1^-*mono*(γ-glutamyl)SPD derivative. Some experimental strategies have made it possible to evaluate the possibility of the activity of an enzyme, the FAD-PAO, involved in the catabolism of polyamines and in particular of the *N*^1^-*mono*(γ-glutamyl)SPD derivative. Indeed, as already reported, under these experimental conditions, the enzyme FAD-PAO is capable of catalyzing the in vitro degradation of peptide-bound *N*^1^-*mono*(γ-glutamyl)SPD and *N^8^*-*mono*(γ-glutamyl)SPM [15]. This catabolic process produces free polyamines and peptide-bound γ-glutamyl-3-aminopropionaldehyde. This could explain the difference in the levels of the two mono-derivatives found in the presence of endogenous SPD and Ca^2+^ in the lens culture medium. To confirm this hypothesis, the activity of lens FAD-PAO was inhibited by MDL 72527, which is a well-known specific inhibitor of this enzyme [32]. Under these experimental conditions, the level of *N*^1^-*mono*(γ-glutamyl)SPD reaches similar levels as the *N*^8^-*mono*(γ-glutamyl)SPD, with the consequent drastic reduction of the formation of *N*^1^-*N*^8^-*bis*(γ-glutamyl)SPD protein cross-link. It is particularly interesting in this context to consider that the increase of both *mono*(γ-glutamyl)SPD derivatives, leads to saturation of the γ-glutamyl sites of the available substrate proteins, thus drastically reducing the possibility of *bis*(γ-glutamyl)SPD cross-links formation, as observed in our experimental conditions. It could also be assumed that the *N*^8^-*mono*-derivative, as a result of the degradation action of the FAD-PAO on the *N*^1^-*mono*-derivative, may have a lower affinity in the attack on the glutamine protein residue. This could be an additional explanation for the drastic reduction in *bis*-SPD cross-links. Work is in progress to find evidence to prove this hypothesis.

## 5. Conclusions

In conclusion, in an experimental cataract model, we report evidence that eye lens opacification, induced by Ca^2+^, derives from the polymerization of *β*B3 crystallins, as a consequence of intraocular TG2 activation. In this model, the increased concentration of endogenous SPD in the lens can drastically reduce crystallins opacification, most likely as a result of saturation of the binding sites of TG2 substrate protein and FAD-PAO activation. The ability of SPD and MDL 72527 to modulate enzymatic activities, involved in the mechanism of lens opacification suggests a potential future strategy for the treatment of senile cataract.

## Figures and Tables

**Table 1:** Levels of (γ-glutamyl)SPDs in the proteolytic digest of cultured rabbit eye lens with or without exogenous SPD.

(γ-Glutamyl)SPD Derivatives (pmol/mg of Lens Proteins)						
Time (h)	72		96		120	
Protein bound derivative	−SPD	+SPD	−SPD	+SPD	−SPD	+SPD
*N^8^-mono*(γ-glutamyl)SPD	12.3 ± 0.5	37.8 ± 2.5	10.2 ± 3.5	68.2 ± 4.3	6.7 ± 0.2	87.7 ± 5.7
*N^1^-mono*(γ-glutamyl)SPD	50.3 ± 3.5	28.7 ± 1.5	36.4 ± 8.7	30.7 ± 5.4	22.6 ± 9.2	15.3 ± 0.7
N^1^,*N^8^-mono*(γ-glutamyl)SPD	65.7 ± 8.4	50.5 ± 2.5	96.4 ± 13.0	37.2 ± 6.0	125.4 ± 14.9	17.4 ± 0.4
Lens transparency %	40	50	30	60	10	80

Quantitative evaluation of the *mono*- and *bis*(ϒ-glutamyl) derivatives of SPD in rabbit eye lens incubated with or without SPD. After 24 h of incubation, medium was supplemented with Ca^2+^ in order to reach a 20 mM and 100 mM concentrations for SPD (see Materials and Methods). Results are expressed as the average of three determinations ± SD.

**Table 2:** Levels of (γ-glutamyl)SPDs in the proteolytic digest of cultured rabbit eye lens upon FAD-PAO activity inhibition by MDL 72527.

(γ-Glutamyl)SPD Derivatives (pmol/mg of Lens Proteins)						
Time (h)	72		96		120	
Protein bound derivative	CTRL	MDL 72527	CTRL	MDL 72527	CTRL	MDL 72527
*N^8^-mono*(γ-glutamyl)SPD	38.3 ± 7.4	36.5 ± 8.3	78.2 ± 3.6	67.2 ± 3.5	98.7 ± 13.8	92.7 ± 14.8
*N^1^-mono*(γ-glutamyl)SPD	25.5 ± 4.5	65.3 ± 6.8	20.8 ± 3.6	78.2 ± 5.4	14.2 ± 0.4	88.7 ± 3.2
N^1^,*N^8^-mono*(γ-glutamyl)SPD	46.4 ± 6.8	76.0 ± 4.7	29.2 ± 2.8	85.3 ± 4.9	15.2 ± 0.6	7.6 ± 0.9
Lens transparency %	50	35	60	30	80	90

Quantitative evaluation of the *mono*- and *bis*(γ-glutamyl) derivatives of SPD in eye lens incubated with or without 400 μM MDL 72527. After 24 h of incubation, medium was supplemented with Ca^2+^ in order to reach a 20 mM and 100 mM concentrations for SPD (see Materials and Methods). Results are expressed as the average of three determinations ± SD.

## Supplementary Materials

Supplementary materials can be found at https://www.mdpi.com/1422-0067/21/15/5427/s1.
