# Supplementary figures and images for: Polyamine Oxidase Is Involved in Spermidine Reduction of Transglutaminase Type 2-Catalyzed βH-Crystallins Polymerization in Calcium-Induced Experimental Cataract
Source: Int J Mol Sci. 2020 Jul 30;21(15):5427. doi: 10.3390/ijms21155427 (PMC7432200; doi:10.3390/ijms21155427)

## Slide 1
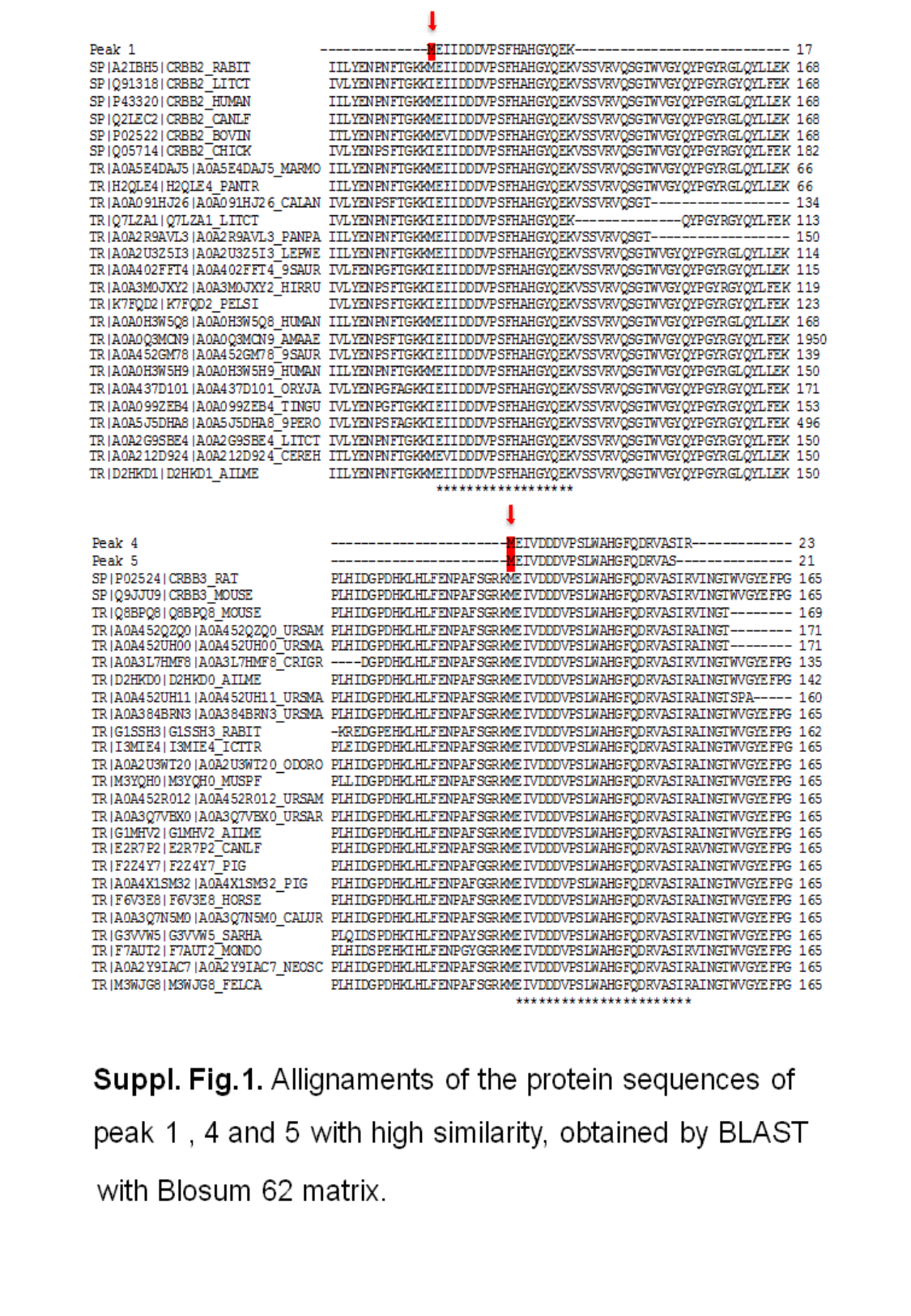

## Slide 2
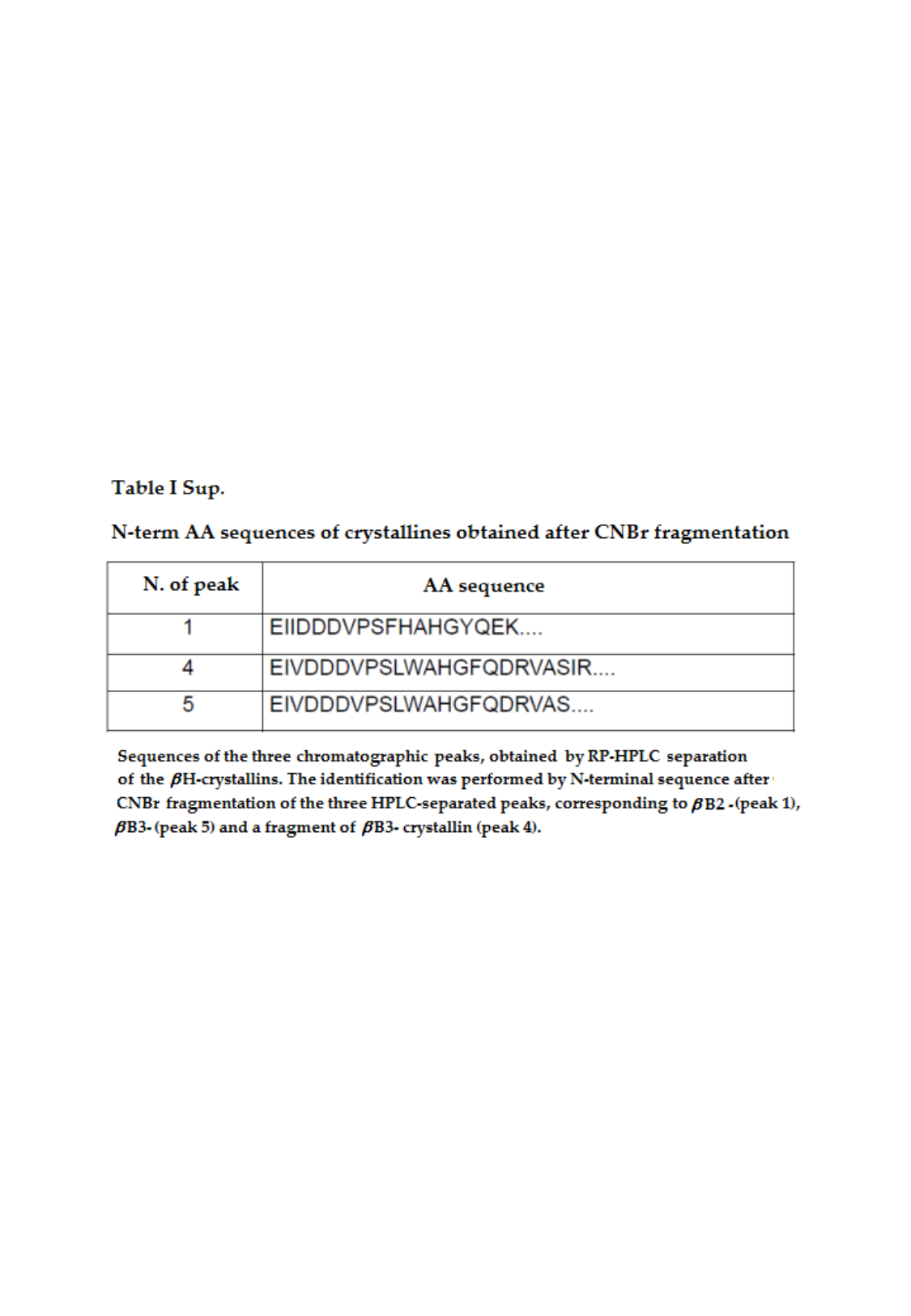

Supplement: Supplementary file 1 [file ijms-21-05427-s001.zip › ijms-876289-supplementary.pptx]
